# Intraocular lens calculation for cataract surgery in high myopia: a case report of an extreme axial eyeball length of 35.7 mm

**DOI:** 10.3389/fmed.2025.1450922

**Published:** 2025-07-04

**Authors:** Andreas F. Borkenstein, Eva-Maria Borkenstein

**Affiliations:** Private Practice at Privatklinik Kreuzschwestern, Graz, Austria

**Keywords:** intraocular lens calculation, high myopia, extreme axial length, anisometropia, biometry

## Abstract

Extreme axial eyeball length is a known risk factor for decreased accuracy of intraocular lens calculations. We report a case of a 35 years-old male with anisometropic amblyopia and an axial length of 35.7 mm in his right eye. To the best of our knowledge, this is a case of one of the longest eyeballs ever scientifically reported. The patient presented with presenile cataracts in the right eye, hand movement visual acuity, and required cataract surgery. Due to his high anisometropia, he was unable to wear spectacle correction prior to surgery in the amblyopic eye. The left (non-amblyopic) eye had moderate myopia and corrected to 20/20 with no signs of cataracts. Various intraocular lens (IOL) calculations were compared, aiming for a slight postoperative residual myopia in the right eye (approx. −1.50 D). The patient required a minus-power intraocular lens, and an IOL with a larger overall diameter (model 92S “Bigfoot IOL” manufactured by Morcher GmbH, Stuttgart, Germany) was selected. On the last available visit at 6 months, his refractive error was −1.50 to −0.75 D × 179° with a corrected visual acuity of 20/63. The large IOL was stable in the capsular bag, and there were no postoperative complications. The patient was able to wear spectacles with correction in both eyes and reported significant improvement in binocular vision and quality of life. Negative power IOLs typically have different optic configurations and require special considerations during IOL calculation. Care should be taken to avoid postoperative hyperopic refractive error. Nowadays, surgeons can choose from a selection of traditional formulas, newer-generation formulas, and axial length adjustment techniques to improve refractive predictability of eyes with extreme axial myopia. With this case report, we would like to demonstrate that even in rare cases of extreme, high myopia and eyeball length > 35 mm, good results can be achieved with correct IOL power implantation if certain considerations are made prior and during the procedure. Special considerations should be taken into account to maximize patient satisfaction.

## Introduction

High myopia, especially extreme axial myopia, poses a challenge for cataract surgery. Special considerations are required when treating such patients. Structural characteristics of eyes with high axial myopia, such as large capsular bag volume or zonular weakness, might increase the instability of IOL and require careful selection of intraocular lens type (1, 2). Not only the higher axial length but other biometric characteristics, such as greater than average white-to-white corneal diameter or greater anterior chamber depth, need to be considered (3). High myopia is also associated with the presence of typical pathologies, such as myopic maculopathy and posterior staphyloma, further complicating the cataract surgery or the accuracy of biometric measurements (4).

The biggest challenge surgeons face is the accuracy of intraocular lens calculation, especially in eyes with extreme axial myopia, requiring the implantation of a “minus” power intraocular lens (5). The main issue is that these cases are relatively rare. Hence, it is almost impossible for surgeons in smaller surgical practices to collect large datasets and retrospectively review their outcomes to determine which intraocular lens formula works the best in these particular cases. For that reason, physicians often rely on the literature reports, but the choice of formulas nowadays is overwhelming (5, 6).

In this paper, we describe our own experience with a patient with extremely high axial myopia and discuss the challenges surgeons face when treating these patients. It should be emphasized that this is an extreme axial length and to the best of our knowledge one of the anatomically longest eyes ever scientifically reported/published.

## Case presentation

A 35 years-old male presented in our clinic with the diagnosis of presenile cataract requiring cataract surgery and a history of amblyopia in the right eye. General medical history included type 1 diabetes mellitus. His axial length in the right eye was 35.70 mm, with an anterior chamber depth of 3.69 mm and keratometry readings of 41.97/43.63 Diopters. The patient had only hand movement visual acuity in his right eye. An auto-refraction measurement was not possible due to the extreme myopia being out of range for measurement with automated devices, and an accurate subjective manifest refraction was difficult to obtain due to poor corrected visual acuity caused by cataracts and amblyopia. His own information from opticians’ examinations from previous years confirmed refraction values of > −20 Diopters.

His left eye had a myopic refractive error (sphere −4.50 D cylinder −1.50 axis 174°) correcting to 20/20 visual acuity. The patient was unable to wear any correction for the right eye due to high anisometropia and only had glasses to correct the refractive error in his left eye, with a balance lens in his right eye. A careful fundus examination was attempted, searching for typical myopic pathology signs, such as fundus myopicus, with typical findings like Fuchs dots, Lacquer cracks, and posterior staphyloma with chorioretinal atrophy. However, an accurate fundus examination was not possible due to the advanced opacity of the lens and limited fundus view. Optical coherence tomography measurement also could not be obtained. The pathophysiology of fundus lesions could only be assessed from an examination performed by an external clinician 6 years prior, before the onset of cataracts.

**FIGURE 1:**
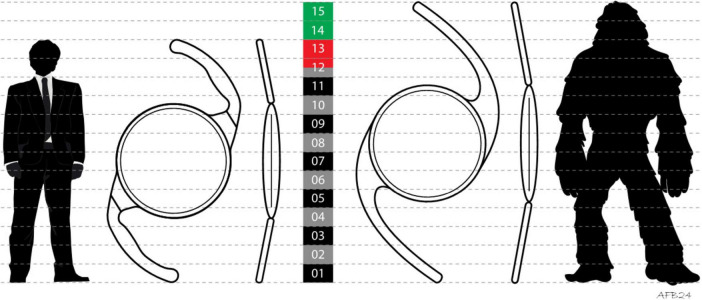
Comparison of a standard one-piece, C-loop intraocular lens (which usually has a total diameter of 12.5–13.0 mm **(left)** and the Morcher type 92S “Bigfoot IOL” **(right)** with a total diameter of 15.0 mm. To illustrate the proportions and in reference to the model’s name, a human man is also compared with the primate Gigantopithecus from the ape family (Hominidae) named Yeti/Bigfoot.

Intraocular lens calculation was performed comparing different formulas, targeting the postoperative spherical equivalent of −1.50 D (Table 1). The aim of the lens calculation was to include publicly available formulas that most surgeons have access to either through web calculators or in biometry devices like the IOLMaster by Carl Zeiss, Germany. The lens selected for implantation was the model 92S “Bigfoot IOL,” manufactured by Morcher GmbH, Stuttgart, Germany. The implanted power of the IOL was −4.0 D, as this power predicted slight postoperative myopia in all our reviewed calculators (Table 1). The model 92S is a monofocal, spherical, one-piece hydrophilic acrylic IOL with 28% water content. The optic diameter is 6.0 mm with an overall diameter of 15.0 mm, making it a good choice for highly myopic eyes with larger capsular bags. The lens is foldable and compatible with clear corneal incisions (CCI) down to 1.8 mm (Figure 1).

**TABLE 1:** Prediction error of different intraocular lens calculation formulas.

IOL calculator/formula name (weblink)	Predicted spherical equivalent with −4.0 D lens (lens model 92S, Morcher GmbH, Stuttgart)	Prediction error (predicted spherical equivalent from the IOL formula minus actual postoperative spherical equivalent)	Comments
**ESCRS calculator^1^** (https://iolcalculator.escrs.org)	---	---	Unable to calculate, AL > 35 mm
**KANE** (https://www.iolformula.com)	---	---	Unable to calculate, AL > 35 mm
**Hill-RBF v3** (https://rbfcalculator.com/online/index.html)	---	---	Unable to calculate, AL > 35 mm
**Barrett Universal II** (https://calc.apacrs.org/barrett_universal2105/)	−0.88 D	0.995 D	
**EVO v2** (https://www.evoiolcalculator.com/calculator.aspx)	−1.42 D	0.455 D	
**SRK/T** (integrated in IOLMaster)	−3.13 D	−1.255 D	
**Holladay 1** (integrated in IOLMaster)	−3.17 D	−1.295 D	
**Hoffer-Q** (integrated in IOLMaster)	−3.60 D	−1.725 D	
**Haigis** (integrated in IOLMaster)	−2.72 D	−0.845 D	

^1^ESCRS stands for The European Society of Cataract and Refractive Surgeons. Formulas available in the calculator: Barrett Universal II, Cooke K6, EVO, Hill-RBF formula, Hoffer QST, Kane, and PEARL-DGS.

**FIGURE 2:**
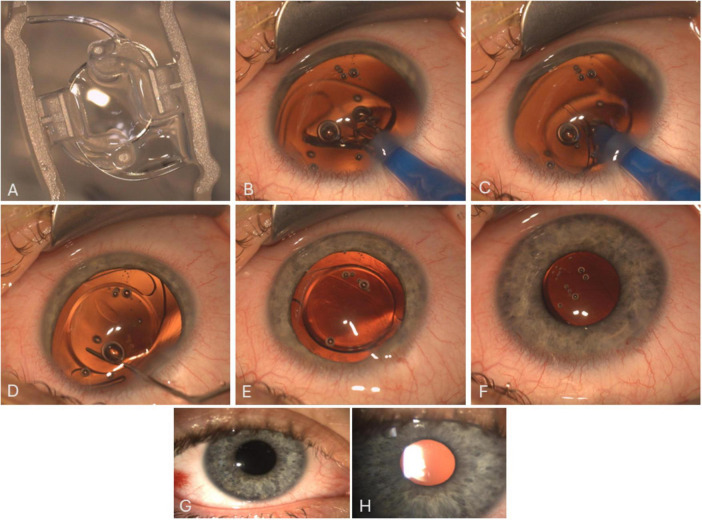
Image **(A)** shows the lens as supplied by the company. Images **(B–F)** are a sequence/snapshots from the OR showing the Morcher 92S IOL and the implantation process. Despite the anatomical conditions (huge eyeball with large capsular bag), a simple, one-step implantation of the Bigfoot IOL through a clear corneal incision (2.4 mm) into the capsular bag is possible. The IOL is then positioned/centered using a spatulum and overall stability is checked finally. Images G and H are slit lamp images showing the well-centered IOL 1 day **(G)** and 4 weeks **(H)** after surgery.

The surgery was performed uneventful. A floppy iris syndrome was found and the patient wasn’t responding well to mydriatic agents and the surgery had to be performed through medium-size pupil. Due to the extreme anatomical length, surgery was challenging as the focus of the microscope had to be adjusted several times. Cohesive and dispersive ophthalmic viscosurgical devices (OVDs) were used and the phaco energy was kept as low as possible. A slow and cautious approach was chosen based on the risk factors of the high myopic eye. The lens was implanted into the capsular bag via a 2.4 mm clear corneal incision without any complications. The large diameter of the lens (15.0 mm) was advantageous for secure positioning of the lens in the large (and floppy) capsular bag (Figure 2).

At the 1 month postoperative visit, his refractive error in the right eye was −1.75 to −0.50 D × 176° correcting to 20/100 (0.7 logMAR). Three months postoperatively, the refractive error was similar (−1.50 to −0.75 D × 179°) with a slightly improved corrected visual acuity of 20/63 (0.5 logMAR), and the refraction/visual acuity remained stable up to the last available visit at 6 months. There were no postoperative complications or IOL stability concerns during the available postoperative course. The examination of the retina showed typical changes due to the extreme myopia but without retinal tears, holes or hemorrhages. The macula showed no edema or other pathology on the optical coherence tomography.

Postoperatively, the patient reported significant improvement in the quality of vision and the quality of life. Over time in the first 6 months, he subjectively perceived a further improvement in the quality of vision and had no neuroadaptation issues. The patient was able to wear contact lenses and glasses with correction in both eyes. Visual fields, contrast sensitivity, color perception, and overall binocular vision improved after surgery. The patient did not meet driving visual requirements before surgery but was also able to pass the medical driving tests 8 months after surgery.

## Discussion

Cataract surgery in the presence of extreme pathologic myopia, such as the one described in this study, is not always straightforward. Firstly, careful planning and preoperative consideration of the pathology of the myopic eye are required. High myopia, younger age, and male gender are the most commonly cited risk factors for retinal detachment after cataract surgery, and our case meets all three of them (7, 8). In high myopes, retinal evaluation with detailed indirect ophthalmoscopy with scleral indentation or even prophylactic treatment to all lesions that contribute to retinal tear is recommended (9). However, such procedures are often complicated by the presence of mature cataracts and limited fundus view. Even though those patients are advised not to delay surgery when visually significant cataracts are present, they often postpone treatment for many years, leading to limited fundus view, reduced visual prognosis, and the possibility of higher complication rates.

Postoperatively, patients should be carefully monitored and vigilant of the signs and symptoms of retinal detachment. Discussion of realistic expectations is another critical preoperative consideration. Our patient had extreme myopia with anisometropia, which is often combined with amblyopia. Thus, realistic expectations and the limits in visual acuity gain need to be discussed. On the other hand, patients with such high anisometropia have difficulties with spectacle correction, and even a slight improvement in corrected vision and reduction of anisometropia is a positive and life-changing outcome in these patients.

Preoperative biometry measurement is another consideration in the treatment of extreme axial myopia. High myopes often have fixation issues, and overall, the measurement of axial length in this particular group of patients is less reliable due to the higher prevalence of ocular co-pathologies. Optical biometry, which is based on partial coherence interferometry, has been found to have good repeatability in patients with high axial length (10, 11). Modern biometers are also capable of measuring other variables in a single measurement, such as the lens thickness, white-to-white, and anterior chamber depth, which are incorporated in some modern IOL calculation formulas.

The most difficult part of preoperative planning, however, is the selection of the correct IOL power. In our experience, aiming for slight postoperative myopia is the best choice. The patient will be more likely to tolerate residual myopia than residual hyperopia. Postoperative hyperopic shift or hyperopic refractive surprise is also not uncommon in high myopes and needs to be accounted for (12–14).

Finding the best IOL calculation formula is not a straightforward task. From the traditional IOL formulas, Haigis (6, 12, 13, 15–21) and SRK/T (12, 18, 21, 22) are commonly cited as reasonably accurate in high myopes. From the choice of newer formulas, Barrett Universal II (6, 15, 17, 19, 20, 23–27), Hill-RBF (Radial Basis Function) (5, 15, 19, 23), Kane (5, 6, 17, 23, 25, 28), EVO (Emmetropia Verifying Optical) (17, 25, 28), XGBoost (Extreme Gradient Boosting) (5, 29) or Olsen (17) have been found to improve refractive accuracy in high myopes. Wang-Koch axial length adjustment methods to traditional formulas have often been recommended in high myopes (6, 17, 19, 23, 27).

**FIGURE 3:**
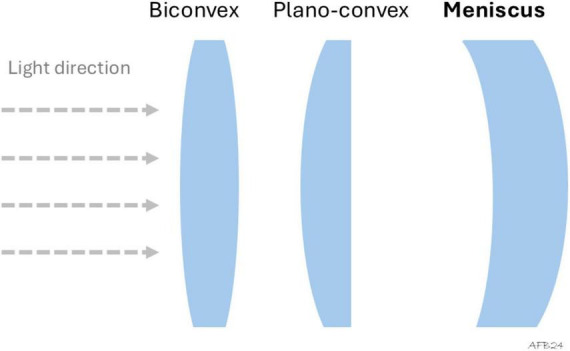
Geometry of intraocular lenses: in biconvex design, the power of the lens is split between the anterior **(front)** and posterior **(back)** surfaces. It is the most commonly used intraocular lens geometry. Meniscus IOLs (convex-concave) are typically used for negative power (minus) intraocular lenses.

However, most of the IOL formula-recommending studies define high myopia as an axial length of 26 mm or more. Very few studies distinguish between the refractive accuracy of patients implanted with “plus power” IOLs and “minus power” IOLs. Negative power IOLs or low plus power IOLs are used in patients with extreme axial myopia and typically have different optic principal planes compared to traditional IOLs (e.g., meniscus-type optic) (Figure 3). Thus, their refractive accuracy should ideally be evaluated separately. Yet, very few studies performed separate analyses of negative power lenses. In 2009, Petermeier et al. (18) compared the IOL calculation accuracy of the lenses in the power range between 5.0 and −5.0 D and found that traditional formulas such as SRK-T, Hoffer-Q, Holladay-2, and Haigis performed reasonably well in negative-power IOLs, but only after the optimization of the A-constants. In another study, Ghanem and El-Sayed (13) found that high myopic eyes with minus power IOLs had a significant tendency toward postoperative hyperopia compared to eyes with plus power IOLs. Of the four evaluated formulas (SRK-T, Hoffer-Q, Holladay-2, and Haigis), Haigis was found to be the most accurate when implanting a minus power IOL. However, unlike most of the recent studies, the IOL calculation was performed using axial length from the immersion ultrasound A-scan (13). Nevertheless, both these studies (13, 18) were published before the newer generation formulas or artificial intelligence-enhanced formulas emerged. Of the more recent papers, the study that focused on the accuracy of plus vs. minus power IOLs in high myopia was the study of Fuest et al. (30). The study found that Barrett Universal II, Haigis, and Hill-RBF had comparable refractive accuracy, but the median absolute error was generally higher in negative power IOLs (which is likely expected when performing surgery in eyes with higher axial length).

In our IOL calculation, we focused on the most commonly available formulas – those that are publicly available in online calculators or incorporated in the IOLMaster by Carl Zeiss Meditec (Table 1). These formulas are accessible to most cataract surgeons. In our particular case, the formula with the lowest prediction error (PE; defined as predicted spherical equivalent from the IOL formula minus actual postoperative spherical equivalent) was EVO v2 (PE = 0.455 D). Haigis and Barret Universal II formulas had a reasonable prediction error within ± 1.00 D. The other formulas from IOL master (SRK/T, Holladay 1, and Hoffer Q) resulted in a higher prediction error, indicating that a standard print-out from IOLMaster or other biometry devices should not be used in such cases of extreme myopia. However, to increase the accuracy of IOLMaster-integrated formulas, axial length adjustment techniques can be applied (31, 32). We also acknowledge that no definitive conclusions can be made about the superiority of any formula from one surgical case. It is important to note that some of the modern lens formulas were out of the range for our patient, accepting only axial length up to 35 mm (Table 1), which limits the choice of IOL formulas in extreme myopia. This fact should also be taken into account in future studies.

The selection of the intraocular lens model in extremely high myopia also needs to be carefully considered. Obviously, the choice of lens models in the minus diopter range is limited. Extreme axial myopia is associated with earlier onset of posterior capsule opacification as well as more severe opacification development (33). IOLs used in these patients need to have PCO-protective features, and their material should be more resistant to possible damage from Nd:YAG laser. These eyes with extreme axial lengths are also at higher risk of vitreoretinal diseases. Hence, the implanted IOLs will be at higher risk of silicone oil exposure during their lifetime (34). Hydrophilic lens materials typically interact well with silicone oil (34). The specific model used in this case report (92S) was found to have low silicone oil adherence (35), low inflammatory response, and good uveal biocompatibility (regarding to manufacturer’s data), making it a good choice for cataract surgery in high myopia. Lastly, the overall geometric design of the IOL needs to be taken into account. Larger capsular bag volume and weaker zonules require an IOL with a larger overall diameter and a robust design, which is another reason for the choice of this particular lens model with an overall diameter of 15.0 mm in our case report.

Lastly, intraoperative and postoperative considerations in cataract surgery for high myopia need to be mentioned. High myopes are at higher risk of intraoperative complications such as posterior capsular rupture, nucleolus drop, or zonular dehiscence (6, 36). Postoperatively, a higher incidence of complications such as retinal detachment, IOL dislocation, refractive surprise, or posterior capsule opacification development has been reported (4, 37). Surgeons should anticipate some of these complications and be prepared to manage them to minimize the potential vision loss. Since the number of such extreme cases is low, multicenter studies should be planned to generate more data and thus enable a better prognosis (with maximum safety and low complications) in these special cases as well. However, individual case descriptions such as ours can point to the particular challenges and show that a well-considered approach can lead to very pleasing results.

## Conclusion

Cataract surgery in patients with extreme axial myopia is complex. Careful preoperative planning and examination are required to minimize future complications and a potential hyperopic surprise. Intraoperatively, the pathology of a myopic eye should be considered, and any complications should be meticulously managed to prevent the risk of further vitreoretinal complications. Despite all these challenges, cataract surgery in patients with extreme myopia often results in life-changing outcomes. The patient in our case report had a disabling degree of preoperative myopia. Reduction of refractive error and an improvement in corrected visual acuity in these patients can make a substantial difference to their quality of life (even in amblyopic eyes with reduced forecast).

## Data Availability

The raw data supporting the conclusions of this article will be made available by the authors, without undue reservation.
